# Prevalence of Noncommunicable Diseases and Their Risk Factors in Guangzhou, China

**DOI:** 10.5888/pcd11.130091

**Published:** 2014-03-27

**Authors:** Bingying Pan, Xiongfei Chen, Xueji Wu, Jinxiang Li, Jipeng Li, Yaohui Li, Xiaommeng Hao, Huazhang Liu

**Affiliations:** Author Affiliations: Bingying Pan, Xiongfei Chen, Xueji Wu, Department of Primary Public Health, Guangzhou Center for Disease Control and Prevention, Guangzhou, Guangdong, China; Jinxiang Li, Jipeng Li, Department of Preventive Disease, Zengcheng Center for Disease Control and Prevention, Guangzhou, Guangdong, China; Yaohui Li, Xiaommeng Hao, Department of Preventive Disease, Luogang Center for Disease Control and Prevention, Guangzhou, Guangdong, China.

## Abstract

**Introduction:**

This article reports on the prevalence of noncommunicable diseases (NCDs) and their risk factors in the city of Guangzhou, China, and shows a trend toward epidemic proportions when municipal data are compared with provincial data.

**Methods:**

We conducted the Guangzhou Community Health Survey in the 12 administrative districts of Guangzhou to learn about NCDs and their risk factors. A community-based, face-to-face survey with a stratified multistage cluster sampling was used. Information was gathered on 27,743 respondents, aged 0 to 108 years, with a male to female ratio of 1 to 1. All participants completed a questionnaire, and those aged 15 years or older had a physical examination. Survey results were compared with the provincial results of the 2002 Guangdong Nutrition and Health Survey (GNHS).

**Results:**

The data were weighted to the respondent’s probability of selection and to the age- and sex-specific population. Prevalence estimate of self-reported NCDs was 16.0%. Hypertension and diabetes were reported as the most important NCDs. Of those who responded, 6.8% reported having more than 2 chronic conditions. The adjusted prevalence of hypertension decreased by 13.3% since 2002. Awareness, treatment, and control of hypertension and diabetes were improved. The estimated prevalence of current smoking decreased, and the prevalence of former smoking increased from 2002. However, the prevalence of overweight and obesity, especially central obesity, increased.

**Conclusion:**

Results were encouraging with regard to hypertension and diabetes. However, the unfavorable trends, especially for overweight, central obesity, and passive smoking, call for additional action.

## Introduction

Noncommunicable diseases (NCDs) comprise mainly cardiovascular diseases, cancers, diabetes, and chronic lung diseases. NCDs caused 36 million deaths in 2008, and nearly 80% occurred in low- and middle-income countries (1). Greater income, more mechanization and industrialization, improved access to food, and urbanization and globalization of unhealthy habits have rapidly changed public nutrition and have increasingly exposed the population to greater risk of chronic disease (2,3).

According to China’s latest National Population Census report, in 2010 more than 665 million people lived in urban areas (4), which would cause a large disease burden. Although government data show that southern China has a lower prevalence of NCDs than does northern China, prevention is still challenging. During 2004 through 2008, data from cause of death surveillance showed that 73% to 80% of deaths in Yuexiu (a part of Guangzhou) were caused by cancer and cardiovascular, cerebrovascular, and chronic lung diseases (5). Similar results were found from the municipal monitoring data from 2003 to 2005 (6). However, objective baseline data on NCDs and community resources were rare in local areas. Although Guangzhou has participated in the Guangdong Nutrition and Health Survey (GNHS) (2002) (7,8), the data did not establish a reliable enough baseline. We report on the prevalence of and risk factors for NCDs in Guangzhou and compare the results of provincial and municipal data on NCDs.

## Methods

The data for our study come from the 2009 Guangzhou Community Health Survey (GCHS). In 2009, Guangzhou had 12 administrative districts with 128 communities, 179 blocks, more than 1,000 residential committees, and more than a million households. (A residential committee is an administrative unit that supplies basic public services such as public health, reemployment, and general affairs.) A 5-stage cluster random sampling plan was used to recruit participants from August through December 2009. The stages of sample selection were as follows: 1) random selection of 1 community in each administrative district, 2) random selection of 1 block within the selected community, 3) random selection of residential committees within selected blocks, and 4) random selection of households within selected blocks. All family members, including children and pregnant women, in the selected households who lived in Guangzhou for more than 6 months completed the survey. The age range was 0 to 108 years. The survey was a face-to-face personal interview. All the study participants gave informed consent, and the study received approval from the Guangzhou Health Bureau. A total of 96 residential committees, 9,600 households, and 28,061 individuals were included in the 2009 GCHS, representing the Guangzhou urban permanent residential population of 9.9 million in 2000 (9). In total, 27,743 questionnaires were completed. The response rate was 98.9%.

To improve the survey response rate, we decided to create posters within communities to introduce this survey in advance. We obtained census data from local police stations and verified that households and all their members were listed before sampling. We selected our study population from residents (including infants) who were registered in Guangzhou census data and who had lived there for more than 6 months. We excluded households that had been vacant for 6 months or longer and those in which family members were registered outside of Guangzhou but were living there for work or for other reasons. All interviewers and examiners were trained according to standardized protocols by physicians who received training specifically for the GCHS 2009. Sampled households who agreed to be interviewed were given telephone appointments in advance. Respondents older than 15 signed a consent form before the interview; guardians signed for respondents who were younger than 15 years or disabled. Respondents aged 15 years or older received health examinations on-site. If any member of a household refused the interview, the household was replaced by the nearest neighbors. The replacement protocol was followed when interviewers were refused 3 times by any family member. Community health centers were required to repeat the procedure by resampling if the replacement rate exceeded 10%. Respondents aged 15 years or older answered questions by themselves. Respondents who were younger than 15 years or who were disabled answered questions through their guardians. All family members were interviewed for this survey. Pregnant women were excluded from questions about prevalence and management of hypertension, diabetes, overweight, and obesity if they had been given a diagnosis of preeclampsia or gestational diabetes.

The 2009 GCHS questionnaire had 3 parts. The first part investigated Guangzhou administrative areas: information on number of institutions, annual revenue, fiscal expenditure, population, and education resources were collected. The second part investigated communities, including geographic features, population, birth rate, and so on. The third part focused on residents and had 4 subparts.

The family interview asked about socioeconomic status (eg, number of family members, house type, annual income). We gathered data on each family member’s sex, birth date, insurance status, and ethnic group. We also collected data on health conditions such as self-reported hypertension, type 2 diabetes, gout, chronic gastritis, nasopharyngitis, corresponding treatments, report of illness during the 2 weeks before the interview, and hospitalization during the previous year.

Individuals aged 15 years or older were interviewed regarding their socioeconomic status (marital status, education level, and occupation); risk factors associated with diseases (hypertension, obesity, and diabetes); and the accompanying underlying risk factors of physical inactivity, poor dietary habits, and tobacco and alcohol use. Questions also covered health care access, hypertension, demographics, physical activity, and nutrition information such as eating fruits and vegetables. In addition, there were multiple choice questions on health knowledge (eg, Do you know hypertension diagnosis criteria?) and awareness of community health service center information. Optional supplemental modules contained sets of questions for specific populations (eg, adults aged ≥60 years; married women aged ≤50 years; pregnant women; lactating mothers; children aged <3 years and 3–7 years; adolescents).

In a physical examination, we measured participants’ height, weight, waistline, hipline, systolic blood pressure, and diastolic blood pressure. Blood pressure measurements were collected in accordance with the 1999 World Health Organization and International Society of Hypertension guideline on hypertension (10). Electronic sphygmomanometers (HEM-7071, Omron Corporation) were used in the survey. All machines were standardized by traditional mercury sphygmomanometer before the interview. Blood pressure was measured mostly at the respondents’ home, but the blood pressure of some respondents was measured at the community health center. Respondents were asked to rest for 5 minutes before blood pressure measurement. Each respondent was measured 3 times with 1 minute of rest between measurements. The 3 measurements were averaged to obtain a reading. Hypertension was defined as follows: 1) an average systolic blood pressure 140 mm Hg or higher, 2) an average diastolic blood pressure 90 mm Hg or higher, or 3) current use of prescribed antihypertensive medicine (self-reported). Hypertension was considered to be controlled if the participant had an average systolic blood pressure of less than 140 mm Hg and an average diastolic blood pressure of less than 90 mm Hg.

Awareness of hypertension was self-reported, defined as any previous diagnosis of hypertension by a health-care professional. Treatment of hypertension was defined as taking antihypertensive drugs within the 2 weeks before the interview. Hypertension was considered as controlled among those on treatment if the average blood pressure was 140/90 mm Hg or lower. Self-reported NCD was defined as any diagnosis by a health-care professional of cardiovascular disease; diabetes; chronic respiratory disease; tumor; or chronic conditions such as gout, chronic gastritis, and nasopharyngitis.

Smoking was defined as currently smoking or having at least 1 cigarette daily for the previous 6 months or longer. Former smoking was defined as cessation of smoking at the time of the interview. Passive smoking was defined as nonsmokers inhaling smoke produced by smokers for a cumulative period of 15 minutes per day for 1 week. Alcohol consumption was defined as drinking at least once per week during the previous 12 months. Physical exercise was defined as more than 90 minutes of moderate physical activity per week.

The weight and height of the participants were measured with light indoor clothing without shoes. Waist circumference was measured horizontally around the smallest circumference between the ribs and the iliac crest. Hip circumference was measured horizontally around the largest circumference of the hip. Both weight and waist were measured between meals. Body mass index (BMI) was calculated as weight in kilograms divided by the square of height in meters.

Normal weight was defined as having a calculated BMI of 18.5 to 23.9 kg/m^2^, overweight was 24.0 to 27.9 kg/m^2^, and obesity was 28 kg/m^2^ or more. Central obesity calculated by waist circumference was defined as 85 cm or more for men and 80 cm or more for women; central obesity calculated with waist to hip circumference ratio (WHR) was defined as 0.86 or more for men and 0.82 or more for women (P.Y. Fu, unpublished master’s dissertation, 2007).

Paper questionnaires were collected 1 week after a 3-level quality control process. In the first level, interviewers and quality controllers checked questionnaires as soon as the interview was completed. In the second level, senior quality controllers from branch center for disease control and prevention checked the questionnaires. In the third level, the highest quality controllers from the Guangzhou Center for Disease Control and Prevention checked all questionnaires. Survey participants were re-interviewed if questionnaires did not meet quality control standards. Missing answers and errors in logic were the most common problems. Eventually, EpiData version 3.0 (EpiData Association, Odense, Denmark) was used to create a database. Within 1.5 months, all data were input twice and crosschecked.

A total of 9,600 families (27,743 children, adolescents, and adults) were selected for this survey. All completed the questionnaire, but only those aged 15 years or older (11,783 male and 12,146 female) had a physical examination. The data were weighted to the respondent’s probability of selection and to the age- and sex-specific population by using current population estimates provided by the 5th National Population Census (Guangzhou) in 2000. The nonresponse information was also incorporated into the weight. All means and prevalences calculated represented the overall estimates for the corresponding population in Guangzhou City. SAS version 9.0 (SAS Institute Inc, Cary, North Carolina) was used for the analyses to account for the complex sampling design and to calculate means, prevalence estimates, standard errors, and 95% confidence intervals.

## Results

There were 27,743 respondents who completed the interview. The age range was 0 to 108 years (average age of 39.4 years). The proportion of the group aged 35 to 45 years (17.5%) was the highest; 13.8% (n = 3,814) of respondents were younger than 15 years. The male to female ratio was 1 to 1.003. About 28.3% of adults had an educational attainment of junior middle school (educated for 9 years); 21.7% of adults aged 15 years or older were unemployed and 71.6% of them were married. The highest proportion of family income per capita per year was 10,000 to 19,999 yuan; 33.7% of participants were in this category.

The overall prevalence of self-reported NCDs was 16.0%. The prevalence for females was 16.9%; for males, it was 15.3%. The top 6 self-reported NCDs were hypertension, type 2 diabetes, chronic rhinitis, lipoprotein metabolism disorders, coronary heart disease, and hyperostosis. The prevalence of cancer was reported as 0.2% (0.17% for males and 0.23% for females). Having more than 2 chronic conditions was reported by 1,878 respondents.

The overall prevalence of hypertension among respondents aged 15 years or older was 11.8%, which includes respondents who self-reported and respondents newly found to have hypertension through this survey’s physical examination. Among people with hypertension, 54.4% were aware of their condition, 49.3% received treatment, and 23.2% controlled their blood pressure. More women than men with hypertension were aware of their condition, and women were more effective at treating and controlling their blood pressure (Table 1).

**Table 1:** Age-Adjusted Prevalence, Awareness, Treatment, and Control of Hypertension Among Respondents Aged 15 Years or Older (N = 27,743) in Guangzhou, China, 2009

Characteristic	Total % (95% CI)	Male % (95% CI)	Female % (95% CI)
Prevalence	11.8 (10.9–12.6)	11.4 (10.5–12.3)	12.1 (11.1–13.1)
Awareness	54.4 (51.7–57.2)	50.5 (46.9–54.0)	58.5 (55.6–61.3)
Treatment	49.3 (46.6–52.0)	44.3 (41.1–47.6)	54.3 (51.3–57.2)
Control	23.2 (20.7–25.7)	22.4 (19.3–25.4)	24.1 (21.7–26.4)

Abbreviation: CI, confidence interval.

**Figure 1:**
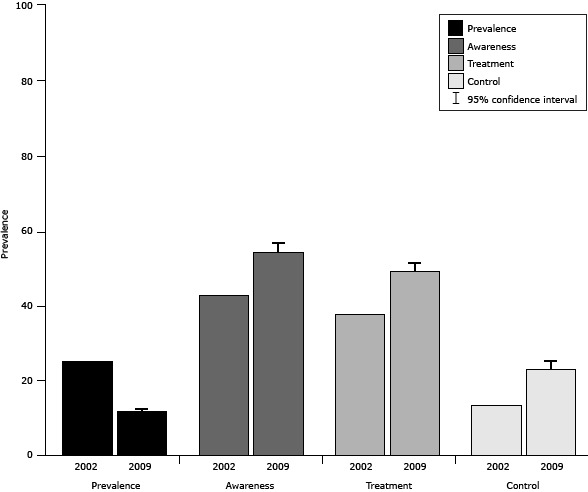
Comparison between provincial (2002) and municipal (2009) results for hypertension prevalence and self-management, Guangzhou, China. Confidence intervals were not available for 2002 data. Respondents in both survey years were aged 15 years or older. The 2002 survey included rural residents of Guangzhou. **Table:** 

The prevalence of hypertension in Guangzhou decreased from 2002 (11.8% vs 25.1%), and the prevalence of hypertension awareness increased (54.4% vs 42.8%), as did treatment (49.3% vs 37.8%), and control (23.2% vs 13.5%) (Figure 1).

Year	Prevalence	Awareness	Treatment	Control
**2002, %**	**25.1**	**42.8**	**37.8**	**13.5**
**2009, % (95% confidence interval)**	**11.8 (10.9–12.6)**	**54.4 (51.7–57.2)**	**49.3 (46.6–52.0)**	**23.2 (20.7–25.7)**

The estimated prevalence of self-reported type 2 diabetes among respondents aged 15 years or older was 1.5%. Female respondents (1.6%) reported more type 2 diabetes than did male respondents (1.3%). The prevalence of pharmaceutical treatment for both sexes was 94.0%.

### Relative risk factors

**Tobacco use.** The estimated prevalence of smoking among respondents aged 15 years or older was 21.0% (Table 2). Male respondents (39.3%) had a significantly higher prevalence of smoking than female respondents (1.4%). The prevalence of current smoking was 18.4% and that of former smoking was 12.4%. The proportion of former smokers was 23.7% for female respondents and 12.0% for male respondents. The prevalence of passive smoking (32.5%) was higher than the prevalence of smoking (21.0%). Female respondents reported more exposure to passive smoking (33.1%) than did male respondents (31.5%).

**Table 2:** Prevalence of Relative Risk Factors for Noncommunicable Disease Among Respondents Aged 15 Years or Older (N = 27,743), Guangzhou, China, 2009

Characteristic	Total, % (95% CI)	Male, % (95% CI)	Female, % (95% CI)
**Tobacco use**			
Smoking	21.0 (20.1–22.0)	39.3 (37.4–41.2)	1.4 (1.1–1.7)
Current smoking	18.4 (17.5–19.3)	34.6 (32.9–36.2)	1.1 (0.8–1.3)
Former smoking	12.4 (11.1–13.7)	12.0 (10.7–13.3)	23.7 (16.8–30.5)
Passive smoking	32.5 (30.1–34.9)	31.5 (28.7–34.4)	33.1 (30.8–35.5)
**Obesity and overweight**			
Obesity	4.5 (4.1–4.9)	4.9 (4.4–5.4)	4.1 (3.6–4.5)
Overweight	18.6 (17.9–19.4)	22.1 (20.8–23.3)	15.0 (14.2–15.7)
Central obesity, W	33.1 (31.5–34.7)	34.6 (32.7–36.4)	31.5 (29.5–33.5)
Central obesity, WHR	62.7 (60.4–65.0)	62.9 (60.4–65.5)	62.4 (59.9–64.9)
**Alcohol use (drinking)**	16.6 (15.1–18.1)	27.4 (25.1–29.7)	5.0 (4.2–5.8)
**Physical activity (moderate exercise)**	61.8 (58.2–65.4)	61.5 (57.7–65.2)	62.1 (58.4–65.7)

Abbreviations: CI, confidence interval; W, waist circumference; WHR, waist to hip circumference ratio.

**Overweight and central obesity.** The prevalence of obesity was 4.5% and overweight was 18.6% among respondents aged 15 years or older (Table 2). Although the prevalence of obesity among male respondents (4.9%) was similar to that of female respondents (4.1%), men had a higher prevalence of overweight (male vs female: 22.1% vs 15.0%). The overall prevalence of central obesity calculated by waist measurement was 33.1% and by WHR was 62.7%; 34.6% of the male respondents had an excessively large waist as did 31.5% of the female respondents. However, the prevalence of central obesity among male respondents calculated by WHR (62.9%) was close to that of female respondents (62.4%).

**Alcohol use and physical activity.** The prevalence of drinking among respondents aged 15 years or older was 16.6% (Table 2). More male respondents (27.4%) drank than female respondents (5.0%). Overall, the estimated prevalence of engaging in moderate physical activity among respondents aged 15 years or older was 61.8%.

**Figure 2:**
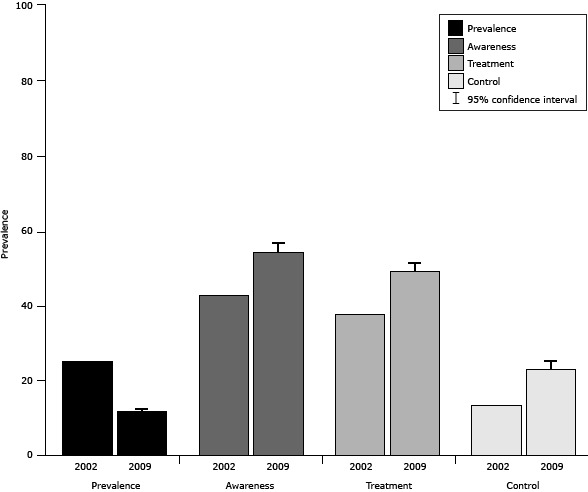
Comparison of provincial (2002) and municipal (2009) results for tobacco use, Guangzhou, China. Respondents were aged 15 years or older. The 2002 survey included rural residents of Guangzhou. **Table:** 

**Comparison of tobacco use between provincial results (2002) and municipal results (2009).** Prevalence of smoking rose by 3.7 percentage points from 2002 to 2009, while current smoking decreased by 1.8 percentage points. However, the prevalence of former smoking also increased sharply by 10.3 percentage points (Figure 2).

Year	Smokers	Current Smoker	Former Smoker
2002, %	17.3	20.2	2.1
2009, %	21.0	18.4	12.4

## Discussion

The prevalence of self-reported NCDs in the city of Guangzhou, China, was 16.0% in 2009, higher than the 13.5% in 1999, according to Guangzhou Regional Planning (2001–2005). The spectrum of NCDs changed during the decade before 2009. Diabetes rose from the sixth most common disease to the second, replacing coronary heart disease.

The prevalence of hypertension among adults aged 15 years or older was 11.8% — a 13.3% decrease from 2002. Moreover, the 2009 Guangzhou result is also lower than that of other urban areas for the population more than 15 or 16 years old around the world (11–15). Only Denmark (16) reported a prevalence of hypertension similar to that of Guangzhou (Denmark, 12.6%; Guangzhou, 11.8%). Dietary salt intake is the likely cause. A recent review (17) shows dietary salt intake in Guangzhou is lower than that in northern China. Management of hypertension has improved since 2002 (18). However, hypertension management remains a larger problem in Guangzhou than in developed areas (19–21). The prevalence of self-reported diabetes mellitus type 2 decreased, but more patients received pharmaceutical treatment (22).

The aging of the population and urbanization are the major forces driving the epidemic of NCDs. In China, Guangzhou had the greatest increase in aging people (people older than 60 years) during 2000 through 2010 (23). Tobacco use, unhealthy nutrition, and physical inactivity leading to obesity and hypertension are already common.

Our survey shows that the prevalence of smoking in other parts of China is higher than in Guangzhou (24). Nevertheless, smoking prevalence in China is higher than that reported for the United States by the Behavioral Risk Factor Surveillance System (2008). Approximately 32.5% of the Chinese respondents reported that they had been or continued to be exposed to passive smoking. The Framework Convention on Tobacco Control in China reported that urban smokers believed that passive smoking can cause lung cancer. The prevalence of smoking was higher than it was in 2002, but the prevalence of current smoking decreased (25). It is encouraging that prevalence of former smoking increased rapidly.

The prevalence of overweight and obesity in Guangzhou is the lowest in China (26). However, an epidemic of central adiposity is imminent; one-third of adults exceed the average waist measurement by an increasing rate of approximately 5.5% (27). The prevalence of central obesity measured by WHR is higher than that in other countries (28–30). Therefore, an effective measurement to control central obesity is urgently needed.

About 61.8% of Guangzhou adults engaged in moderate physical activity (at least 90 minutes per week). The results are similar to the reports of the Population Physical Condition and Research of Guangzhou (2000–2005). The prevalence of alcohol use increased from 2002 to 2009 (31).

In this study, we compared the prevalence of NCDs shown in provincial data with those shown in municipal data and found a rising trend in NCD prevalence since 2002. Establishing a database of factors related to public health in Guangzhou communities is an important step in the effort to reduce the prevalence of NCDs. The advantages of this survey are as follows:

1. A large study size of 28,061 individuals from 9,600 households produced a clear demographic picture of Guangzhou’s urban permanent residential population.
2. The respondent rate is 98.8%, which was higher than that of the China National Nutrition and Health Survey (90%) (32) and that of the New York City Health and Nutrition Examination Survey (55%) (21).
3. The results were used to estimate the number of people with NCDs to determine whether NCD management is achieving stated goals at each community health service center. Furthermore, reducing the prevalence of NCDs has been a large part of China’s medical reform since 2011.

Some limitations of this study should be noted. First, in this survey, few respondents showed the interviewer their medications. Hence, recall bias is inevitable, which may have resulted in overestimating or underestimating prevalence and treatment of NCDs. Second, confounding bias of age was difficult to assess because of the study’s purpose, structure, and length. Because age and sex are important risk factors for NCDs, our next analysis will focus more scientifically on matching NCDs with age and sex.

Results were encouraging in some areas; for example, the prevalence of hypertension decreased and management of NCDs improved. However, the unfavorable trends for most major risk factors (especially overweight, central obesity, and passive smoking) pose an enormous challenge and require additional and timely action and policies.
